# Towards a Stochastic Paradigm: From Fuzzy Ensembles to Cellular Functions

**DOI:** 10.3390/molecules23113008

**Published:** 2018-11-17

**Authors:** Monika Fuxreiter

**Affiliations:** MTA-DE Laboratory of Protein Dynamics, Department of Biochemistry and Molecular Biology, H-4032 Debrecen, Hungary

**Keywords:** protein dynamics, conformational heterogeneity, promiscuity, fuzzy complexes, higher-order structures, protein evolution, fuzzy set theory, artificial intelligence

## Abstract

The deterministic sequence → structure → function relationship is not applicable to describe how proteins dynamically adapt to different cellular conditions. A stochastic model is required to capture functional promiscuity, redundant sequence motifs, dynamic interactions, or conformational heterogeneity, which facilitate the decision-making in regulatory processes, ranging from enzymes to membraneless cellular compartments. The fuzzy set theory offers a quantitative framework to address these problems. The fuzzy formalism allows the simultaneous involvement of proteins in multiple activities, the degree of which is given by the corresponding memberships. Adaptation is described via a fuzzy inference system, which relates heterogeneous conformational ensembles to different biological activities. Sequence redundancies (e.g., tandem motifs) can also be treated by fuzzy sets to characterize structural transitions affecting the heterogeneous interaction patterns (e.g., pathological fibrillization of stress granules). The proposed framework can provide quantitative protein models, under stochastic cellular conditions.

## 1. The Structure-Function Paradigm

Protein functions take place in space and time. Structure-function principles, however, relate a protein sequence to biological activity, only via the spatial coordinates of the residues [1,2]:

SEQUENCE → STRUCTURE → FUNCTION          (*x,y,z*)    (*x,y,z,t*) (1)

The three-dimensional organization of amino acids brings different chemical groups into proximity [3,4], creating specific microenvironments for biological activities. The emerging active sites, for example, can catalyze chemical reactions at significantly faster rates, than in solution [5,6]. The classical, deterministic Paradigm 1 establishes an unambiguous connection between the protein sequence and its function, via a unique structure.

## 2. The Ensemble View

The energy landscapes of proteins are, in reality, more complicated. Proteins fluctuate among various conformations (‘macrostates’) and sub-states (‘microstates’), which need to be considered for their relevant functioning [7,8,9]. A wide spectrum of dynamical transitions [10,11]—from local movements (e.g., sidechain rotations [12]) to large-amplitude collective motions (e.g., domain repositioning [13])—generates conformational ensembles, which, however, are not trivial to link to the function. How can structure-function relationships account for protein dynamics? If protein structure is described as an ensemble, the populations of the relevant sub-states, as well as the rate of interconversion between them, must be experimentally determined for each biological activity.

SEQUENCE → CONFORMATIONAL ENSEMBLE → FUNCTION               {*p*_*cs*_1__,…,*p_cs_N__*}      (*x,y,z,t*)     {*k*_*cs*_1__,…,*k_cs_N__*} (2)

where $p_{CS_{i}}$ is the probability of the given conformational sub-state *CS_i_*, *N* is the number of sub-states, and {$k_{CS_{i}}$} is the set of rates, corresponding to the conversions between *CS_i_*→*CS_j_*, where *j* corresponds to all the other sub-states. Even if the number of conformational states is reduced to a few functionally relevant ones, characterizing both their thermodynamic and kinetic properties is a daunting task [14,15]. Furthermore, the deterministic relationship between the ensemble parameters and a unique function is also influenced by the environmental conditions. 

## 3. Adaptation to Stochastic Cellular Conditions

Proteins function under rapidly changing extracellular signals and intracellular milieu, which is shaped by cellular diffusion and transport, stochastic gene expression, degradation, and other environmental fluctuations. These factors present stochastic conditions for protein evolution [16,17,18] leading to ‘noise’ in biological innovations [19], which is reflected by redundancies and ambiguities in sequences [20], structures [21], and functions [22]. On the one hand, proteins attempt to minimize functional noise. For example, higher-order structures emerge to reduce noise-to-signal ratio for low-affinity substrates [23,24,25]. On the other hand, ambiguities and redundancies in sequence, structure and function facilitate dynamic adaptation [26]. Proteins evolve under these two opposing constraints to optimize fitness under given cellular conditions.

## 4. Ambiguity and Redundancy in Sequence, Structure, and Function

The re-formatted paradigm (2), still implies that a given sequence generates a well-defined ensemble, which belongs to a specific function. The stochastic cellular conditions lead to the following observations, which violate the classical paradigm: (i) A considerable proportion of proteins exhibit multiple, simultaneous activities, often referred to as promiscuity or moonlighting [27]. (ii) Certain biological activities (i.e., signaling) are related to heterogeneous conformational ensembles, which are mixtures of different functional ensembles [28]. (iii) Some proteins exhibit a weak sequence dependence, i.e., a large degree of tolerance towards sequence modifications [29]. These observations stem from redundancies in sequence or structure, coupled to ambiguities in function. The same ensemble may perform multiple functions (*functional promiscuity*); the same sequence may be organized into multiple functional ensembles, depending on the context (*conformation and interaction heterogeneity*); and multiple sequences may encode the same conformational ensemble (*sequence redundancy*). These problems, which reflect a more complex relationship between the sequence, structure, and function of proteins, are detailed below.

## 5. Functional Promiscuity

**Figure 1:**
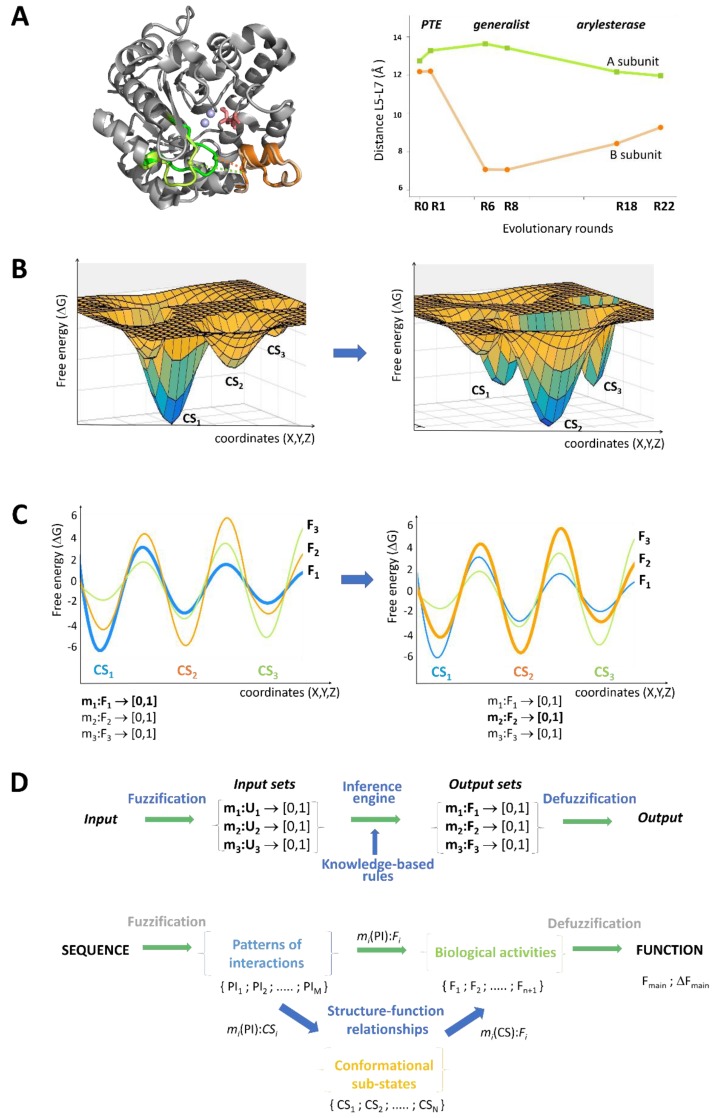
Towards a stochastic structure-function relationship. (**A**) Structural diversity increases with functional promiscuity. The distance between the L5 (*lime*, *green*) and L7 (*wheat*, *orange*) loops (A204 C–G273 C) deviates in the two subunits (*superimposed*) of a dimeric phosphotriesterase (PTE) enzyme (PDB code: 4xag [39]). During laboratory evolution into arylesterase, the structural difference increases as the two activities become comparable (R1 → R6), while it decreases during specialization (R8 → R22). (**B**) Free energy landscape changes upon adaptation of proteins. Functional alterations shift the relative populations of conformational sub-states, but may not impact the ruggedness of the landscape. (**C**) Conformational sub-states (CSs) contribute to multiple free landscapes. The functional noise (uncertainty of F_1_, F_2_, F_3_) of the main activity (*bold*) can be quantified by fuzzy membership functions. (**D**) The fuzzy structure-function model. In the fuzzy inference system, the logical relationship is established between the fuzzy sets of the input and output (*top*). In proteins, fuzzification generates sets of interaction patterns amongst functional sequence motifs, which can be linked to conformational sub-states. The connection between structure and function is a knowledge-based logical rule between the set of conformational sub-states and the set of alternative functions, from which the most likely activity can be selected (*bottom*).

Metabolic enzymes often catalyze reactions on the non-canonical substrates, some of which are also relevant physiologically [27,30,31]. Functional promiscuity may parallel organism complexity [32], or be driven by network context [33]. Promiscuous activities can serve as starting points to engineer new enzymes [34]. Tailored selection pressures may optimize latent activities to overcome the primary function by >10^9^-fold [35]. Functional transitions are usually initiated by ‘neutral drifts’, with a negligible impact on the original activity [36,37]. That is, the optimization of a promiscuous function initially exploits the inherent variations in structure [38] and dynamics [39]. Functional transition of a phosphotriesterase to arylesterase [35], for example, is coupled to increasing structural divergence between the two subunits, until the two activities become comparable (Figure 1A). In contrast, specialization for the new activity is accompanied by structural convergence (Figure 1A). Similarly, ‘freezing’ out unnecessary motions offers another route to optimize enzymatic efficiency [6]. Along these lines, principal modes derived from structure [40] often presage or follow the evolutionary changes [41,42].

## 6. Conformational Heterogeneity

Dynamic signals perturb conformational ensembles by changing the relative populations of the different sub-states [43] (Figure 1B). The co-existence of functionally different conformations, in a broad regime, may enable the same protein to be simultaneously engaged in multiple pathways [44]. An agonist binding to a β_2_-adrenergic receptor, for example, does not stabilize the active conformation of the cytoplasmic domain; it rather increases the conformational heterogeneity of the active, intermediate, and inactive states, for the complex signaling outputs [28].

Intriguing observations indicate that specific biomolecular recognition can also be achieved in heterogeneous conformational ensembles [45,46,47]. Although the underlying molecular forces are often puzzling [48,49], conformational ambiguities often enable context-dependent responses, via alternative interaction patterns [50,51]. Conformational heterogeneity along the binding trajectory, has recently been concluded to critically influence the structures in a complex, with different partners [52,53]. Structural ambiguities might even be a pre-requisite, for example, for efficient transcription [54] via a fuzzy ‘free-for-all’ mechanism [55].

Conformational heterogeneity often leads to dynamic interaction profiles, where the functional output (specificity, signal, and polymerization) is controlled by transient contacts [56,57]. Dynamic interactions may also balance between the auto-inhibited and active states [58,59] and can be significantly influenced by post-translational modifications (PTMs) [60,61]. Although the modification pattern inducing the functional response can be defined, its impact on the underlying heterogeneous conformational ensembles often remains unclear.

## 7. Redundant Sequence Motifs

Multiple, weakly-restrained sequence motifs are frequently distinguished in signaling pathways, via mediating protein interactions [62]. Regions linking the motifs exhibit increased conformational plasticity and reduced sensitivity to mutations or scrambling [63], leading to a phenomenon, often referred to as ‘sequence independence’ [64]. Tandem repeats of a few residues, for example, are often involved in the organization of higher-order structures [65], ranging from amyloids to signaling complexes and nuclear pores [66]. Motif redundancy leads to the redundancy of interaction patterns and the co-existence of different contact topologies. Although the interactions of the individual motifs are often sub-optimal, their cooperativity may result in high-affinity associations [25,67].

Both the dynamics of the motif-linking regions, and the variations in contact patterns, lead to conformational heterogeneity in higher-order assemblies [68]. The Fused in Sarcoma (Fus) protein, for example, is involved in the formation of stress granules, via a liquid–liquid phase transition, which is driven by its low-complexity (LC) domain, composed of 27 [S/G]Y[S/G] repeats. The NMR spectra of the LC domain in the droplet, is similar to that of the unbound state, witnessing conformational heterogeneity in the assembly [69]. Single-point mutations may gradually decrease conformational heterogeneity, leading to pathological aggregation [70]. Additional studies corroborate the finding that pathological mutations initially induce minor perturbations [71], which simultaneously affect multiple conformations/interaction patterns and induce their shift towards the fibril form.

## 8. Generalized Structure-Function Ensembles

The experimental data summarized in the above three sections are difficult to interpret via the classical structure-function paradigm (2). We may attempt to solve these problems by treating the sequences, conformations, and functions as generalized ensembles:

SEQUENCE (μ, σ) →CONFORMATIONAL ENSEMBLE (μ, σ) →FUNCTION (μ, σ)(3)

where μ is the mean, and σ is the variance of the respective distribution.

Evaluating the structure-function paradigm in the form (3), requires decoupling of all the respective activities, to analyze the underlying distributions of conformational ensembles and sequences. Careful experimental studies, along these lines [72], demonstrate that these approaches are hardly feasible. First, because the dimensionality of the problem is overwhelming, and second, the deconvolution of different functionalities may not be possible in vivo, owing to the intricate connections.

## 9. Fuzzy Sets Quantify Sequence and Conformation Ambiguities

I propose that the fuzzy set theory [73] offers a quantitative framework to derive stochastic structure-function relationships. In fuzzy sets $U=\left\{x_{1},x_{2},\;...,\;x_{N}\;\right\}\;$ a membership function $m\left(x_{i}\right)\;\to \;\left[0,1\right]\;$; $x_{i}\in \;U$ is assigned to each element, which characterizes to what extent $x_{i}$ belongs to the given set. For example, the membership of a protein conformational sub-state $\left(CS_{i}\right),$ in a specific functional set ($F_{1}$), can vary between 0 and 1 ($m_{1}\left(CS_{i}\right):\;F_{1}\to \left[0,1\right]$), allowing the conformation to contribute to additional activities (e.g., $F_{2}$ and $F_{3}$, Figure 1C). Memberships for other possible biological functions could also be defined, using this formalism (Figure 1C). In a similar manner, memberships of sequences in given conformational ensembles, ($m_{1}\left(SEQ_{i}\right):\;CS_{1}\to \left[0,1\right]$), or in given functions ($m_{1}\left(SEQ_{i}\right):\;F_{1}\to \left[0,1\right]$), could also be quantified.

The structure-function paradigm could thus, be reformulated by treating the sequences and conformational ensembles as fuzzy sets:

SEQUENCE → CONFORMATIONAL ENSEMBLE → FUNCTION(4)

$$\begin{array}{ll} \;m_{i}\left(\mathrm{PI}\right):{\;\mathrm{CS}}_{i}\to \left[0,1\right] & \;m_{i}\left(\mathrm{CS}\right):{\;F}_{i}\to \left[0,1\right]\\ \;m_{i}\left(\mathrm{PI}\right):{\;F}_{i}\to \left[0,1\right] & \end{array}$$

where $m_{i}\left(PI\right)$ is the respective membership function of a sequence, defined with respect to the conformational states ($CS_{i}$) or biological activity ($F_{i}$), as a pattern of interacting elements/motifs (*PI*). $m_{i}\left(CS\right)$ is the membership function of the conformational sub-state/ensemble (*CS*), in a given function.

Here sequence, structure, and function are considered as different co-existing distributions (Figure 1C), and their contributions change according to the cellular conditions. For example, in the case of a β_2_-adrenergic receptor, the active, intermediate, and inactive states (represented by three ensembles) are mixed differently, depending on the signaling input. The fuzzy formalism handles combinations of activities aiming to determine the individual contributions of the different conformational ensembles.

## 10. The Stochastic Structure-Function Relationship

Within this framework, the structure-function relationship can be quantified by a fuzzy inference system [74,75] (Figure 1D). Parameters describing the elements of the sequence (motifs) or conformational space (distinguished secondary structures) are used as the input, and the different biological activities serve as the output of the system. The first step is the fuzzification of the input, when the fuzzy sets and their membership functions are defined to describe the interaction patterns, and the corresponding conformational sub-states (Figure 1D). The fuzzy inputs are then combined and knowledge-based logical rules (‘IF-THEN’) are applied to obtain the output membership functions of the different biological activities in the system. These rules could be derived using machine-learning or neural network algorithms. Defuzzification of the output can select the most likely activity, under a given condition, while also accounting for other, promiscuous activities (Figure 1D).

The fuzzy model quantifies the functional ambiguities of the conformational sub-states:

(5) $$\;\Delta F_{main}=(\overset{n}{\underset{i}{\sum }}\delta F_{i,main\;})/n\;$$

where *n* is the number of alternative (promiscuous) activities, and $F_{main}$ is the main function with membership function $m_{max}$. The contribution of function $F_{i},$ with respect to the main function, is computed from the corresponding membership functions: $\delta F_{i,\;main}=m_{i}/\;m_{max}$.

Here, the challenge is to define the membership functions. To this end, the efficiencies of the alternative activities (e.g., catalytic rates) are determined via functional assays on well-characterized conformations (e.g., crystal structures, chip-bound proteins, or those selected by conformational antibodies) or ensembles (solution techniques, NMR, FRET, and single-molecule methods). Different membership functions could be probed computationally, based on the regulatory characteristics (e.g., changing an auto-inhibited to an active state).

The fuzzy formalism (4) is particularly useful to relate the sequence sets to function. Here structural features, which could be predicted from the sequence (e.g., secondary structure elements, disordered regions, or post-translational modification sites) may serve to generate the pattern of interaction elements (PI), to define the fuzzy sets. This approach has been implemented in simulations of higher-order protein organizations [76].

## 11. Conclusion and Outlook

Proteins deal with uncertain information, regarding cellular conditions. The information is not only imprecise, but various components are unknown or are unpredictable, owing to the non-random fluctuations in the system. The functional characteristics of proteins need to be adjusted to this poorly defined environment. The classical models in protein science, such as the structure-function paradigm, are based on well-defined properties and cannot deal with the ambiguities related to “noise”. The fuzzy set theory offers a quantitative framework to reformulate the structure-function paradigm for describing the stochastic cellular behavior of proteins (Figure 1D). This approach will provide a more holistic protein model, which can be applied to generate interaction or metabolic networks of different cell lines as well as more reliable pharmacophore models.
